# Cardiac Troponin I Levels in Hospitalized COVID-19 Patients as a Predictor of Severity and Outcome: A Retrospective Cohort Study

**DOI:** 10.7759/cureus.14061

**Published:** 2021-03-23

**Authors:** Jabar Ali, Fahad R Khan, Rizwan Ullah, Zair Hassan, Safi Khattak, Gul Lakhta, Nooh Zad Gul, Rahman Ullah

**Affiliations:** 1 Cardiology/Interventional Cardiology, Lady Reading Hospital, Peshawar, PAK; 2 Cardiology, Lady Reading Hospital, Peshawar, PAK; 3 Cardiology, Lady Reading Hospital, Peshwar, PAK; 4 Gynecology and Obstetrics, Lady Reading Hospital, Peshawar, PAK

**Keywords:** covid-19 pandemic, covid-19 virus disease, sars-cov-2 infection, covid-19 outbreak, covid 19, cardiac troponin i, troponin i, troponin-i and d-dimers, crp levels

## Abstract

Introduction

The COVID-19 (coronavirus disease) has affected millions of people, wreaking havoc worldwide. World Health Organization (WHO) labelled this disease as a serious threat to public health since its rapid spread from Wuhan, China. The respiratory manifestations of COVID-19 are common, but myocardium involvement causing myocardial injury and rise in cardiac markers is much less discussed.

Materials and methods

We conducted this retrospective cohort study from 1st April 2020 to 1st October 2020. Data was collected from the Hospital Management and Information System (HMIS) based on inclusion criteria. We used the Cox proportional hazard regression model for survival analysis, estimated the probability curves of survival using the Kaplan-Meier method, and contrasted it with the log-rank test.

Results

Among the 466 patients, 280 (69%) were male; the rest were female. The majority were both hypertensive and diabetic, and one-third had a myocardial injury on arrival. The most frequent symptoms in more than half of the patients (51.90%) included a combination of fever, dry cough, and shortness of breath. Out of 466 patients, 266 patients were discharged, and 200 did not survive. In our study, 168 (36.05%) patients had a cardiac injury; among them, 38 (22.61%) were in the discharge group, and the remaining 130 (77.39%) patients were in the nonsurvivor group. Our study results showed that the mortality rate was higher in patients with high cardiac troponin I (cTnI) levels (hazard ratio [HR] 3.61) on admission.

Conclusion

Our result concluded that measuring cTnI levels on presentation could help predict the severity and outcome in COVID-19 patients. It will allow physicians to triage patients and decrease mortality.

## Introduction

The pandemic of COVID-19 (coronavirus disease) is the major public health concern of the century. For several decades to come, this outbreak’s health, financial, and cultural consequences will be experienced by human beings [1]. The virus that caused this disease outbreak in 2019, known as SARS-CoV-2 (severe acute respiratory syndrome coronavirus-2), is much more infectious than severe acute respiratory syndrome (SARS) and Middle East respiratory syndrome coronavirus (MERS-CoV) [2]. Besides its impact on the lungs, the novel coronavirus can invade several vital organs (kidneys, brain, and heart), trigger cytokine storm (acute hyperinflammatory response associated with the release of various inflammatory cytokines), followed by multiple organ failure. Of these organs, the heart is a critical target [3]. A literature search showed viral myocarditis and myocardial damage as perpetrators and one of the leading causes of death by COVID-19 [3].

There is ample evidence of COVID-19-related rise in cardiac troponin T and cardiac troponin I (cTnI) beyond the 99th percentile reference point in the literature [4]. The severe cardiac injury is defined as when the cTnI serum concentration exceeds the upper reference point (0.4 ng/ml) three times [5]. Studies have shown that COVID-19 patients with raised cTnI levels had poor prognosis. The risk of ventilation support and intensive care unit need rises by up to five times [6]. Literature is available but scarce on this topic. The current study is the first retrospective cohort study from Pakistan conducted on the COVID-19 infected patients. It established a relationship between cTnI levels and disease severity and outcome and validated findings of earlier studies. It will set a path for other researchers to follow suit to understand better the predictive value of cardiac biomarkers and myocardial damage in COVID-19.

## Materials and methods

Study Population

We conducted this study on all confirmed consecutive COVID-19 patients presented to our hospital between 1st April 2020 to 1st October 2020. We labelled patients as COVID-19 according to interim guidelines from the World Health Organization. This study proceeded after approval from the Lady Reading Hospital’s Ethical Review Board (ERB/REF#585).

Data collection

We retrieved the retrospective records of 466 patients from the hospital management information system (HMIS). After informed consent from patients, we included them based on the inclusion and exclusion criteria. Variables collected were demographics, presenting complaints, comorbidities, laboratory investigations, patients’ outcome (death or discharge from hospital), and time to the event. The reference level for normal cTnI level in our hospital is less than 0.4 ng/ml. Among the laboratory investigations were the baseline investigations (white blood cells [WBC]), acute phase reactants (serum ferritin, C-reactive protein [CRP], and D-dimers), and cardiac troponin I level (cTnI).

Study participants

Our study’s inclusion criteria included patients who had a positive real-time polymerase chain reaction (RT-PCR) test and were older than 18 years of age. Patients presenting with acute decompensated heart failure (ADHF), hypertensive crisis, and cardiovascular accidents (CVA) were not included in the study. These can cause a pseudo rise in cTnI. COVID-19 patients with a history of coronary revascularization either through percutaneous intervention (PCI) or coronary artery bypass graft (CABG) surgery were not enrolled in the study. Patients with severe valvular disease, rhythm disorders such as tachyarrhythmia or bradyarrhythmia, implanted pacemaker, chronic obstructed lung disease (COPD), chronic kidney disease (CKD), along with those who did not undergo cTnI testing at admission were excluded.

Statistical analysis

We performed descriptive analyses after stratifying cTnI levels into normal (0.00-0.4 ng/ml) and elevated levels, i.e., >0.4 ng/ml). We then presented continuous variables as mean and standard deviation and categorical variables as frequency rates and percentages. After that, we applied the Mann-Whitney U rank-sum test for testing the mean values of continuous variables and fisher’s exact test for categorical variables. We then applied multivariate regression using the Cox Proportional Hazards Model to determine the independent risk factors of mortality during hospitalization. We used this model with and without adjusting for other variables. These variables included demographics (age, sex), underlying comorbidities, clinical variable (Body Mass Index [BMI]), and laboratory measurements (serum D-dimers, serum ferritin, cTnI levels, WBCs, and CRP). After that, we estimated the probability curves of survival using the Kaplan-Meier method and contrasted them with the log-rank test.

## Results

During the study period from 1st April till 1st October 2020, we included 466 patients. The patients’ median age (SD) was 55.01 ± 13.49 years (range 22-90 years), and the mean BMI was 24.94 ± 4.81. Among these patients, 280 (60.09%) were male, and 186 (39.91%) were female. The most common comorbidities were diabetes mellitus and hypertension (n = 80, 17.17%). The most common symptoms were dry cough, fever, and shortness of breath (n = 242, 51.93%). A total of 266 (57.08%) survived, and we discharged them, while 200 (42.92%) died. cTnI was raised in 168 (36.1%) patients. 38 (22.61%) were in the survivor group, whereas 130 (77.39%) were in the nonsurvivor group. Besides cTnI, we measured WBCs and acute phase reactants such as serum ferritin, CRP, and serum D-dimer levels in every patient. These baseline line demographics, clinical characteristics, and the patients’ investigations are given in Table 1.

**Table 1:** Baseline demographic and clinical characteristics of the patients n-frequency; %-percentage; DM-diabetes; HTN-hypertension; IHD-ischemic heart disease; cTnI-cardiac troponin I; CRP-C-reactive proteins

Variable	n	%
Gender		
Male	280	60.09
Female	186	39.91
Symptoms		
Dry Cough	64	13.73
Fever	90	19.31
Shortness of breath	70	15.02
Dry cough, fever, and shortness of breath	242	51.93
Cardiac Troponin I (cTnI) levels		
Normal cTnI level	298	63.95
High cTnI level	168	36.05
Serum D-dimer		
Normal	90	19.31
More >400	103	22.10
more >1000	273	58.58
Comorbidities		
DM	16	3.43
HTN	47	10.09
DM and HTN	80	17.17
IHD	74	15.88
No Comorbidities	249	53.43
Serum Ferritin		
Normal	64	13.73
High (more than 200)	160	34.33
very high (more than 1000)	242	51.93
C-reactive proteins (CRP)		
Normal	60	12.88
High	406	87.12
Outcome		
Discharge	266	57.08
Death	200	42.92

A two-tailed Mann-Whitney two-sample rank-sum test was conducted to examine significant differences in age and BMI among patients who died and those who were discharged. The result of the Mann-Whitney U test was not significant for age(p=.198), whereas significant for BMI (p<.001). This suggested that the BMI distribution for the discharge category differed significantly from the BMI distribution for the death category. We then compared categorical variables among groups using the fisher’s exact test.

Unadjusted Cox proportional hazards model

An unadjusted Cox proportional hazards model was conducted to determine whether cTnI, on admission, had a significant effect on the hazard outcome without adjusting for other variables. The outcome discharge category was used to show survival, while the death category represented a hazard event. The coefficient for the unadjusted high category of cTnI was significant; HR = 6.09, p<.001, indicating that observation in the high cTnI category will have a hazard, i.e., 6.09 times as large as the normal cTnI levels category shown in Table 2.

**Table 2:** Unadjusted Cox proportional hazards regression coefficients for troponin I CI-confidence interval; HR-hazard ratio; B-unstandardized beta; SE-standard error; z-ratio of regression coefficient to its standard error

Variable	B	SE	95% CI	z	p	HR
High troponin I	1.81	0.16	[1.50, 2.11]	11.62	< .001	6.09

Adjusted Cox proportional hazards model

**Figure 1:**
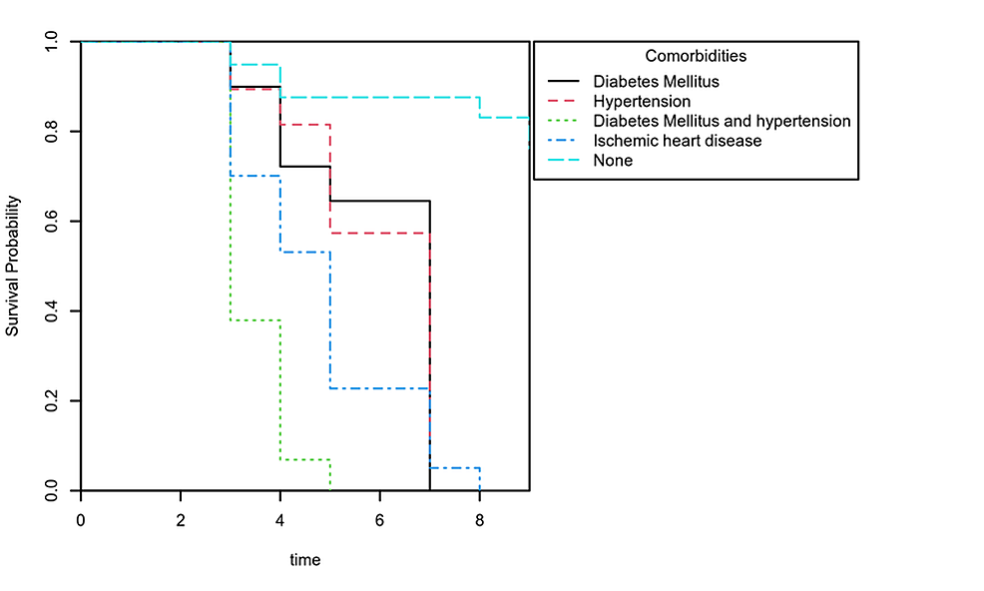
Kaplan-Meier survival plot of outcome grouped by comorbidities

**Figure 2:**
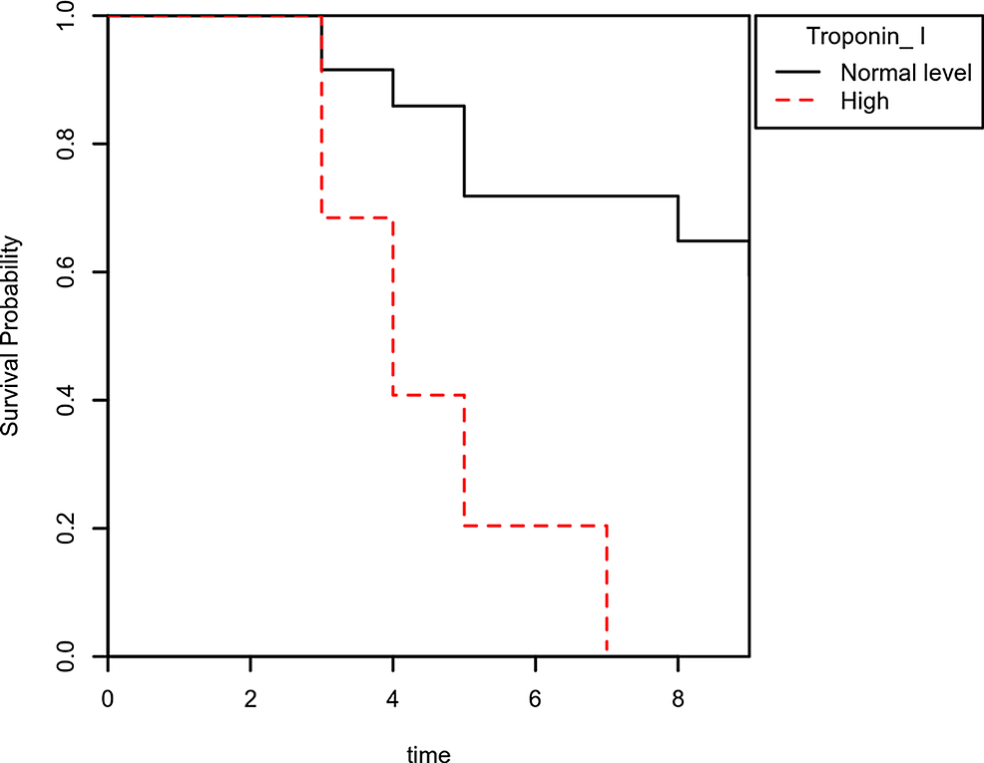
Kaplan-Meier survival plot of outcome grouped by cardiac troponin I (cTnI) levels

We then applied the Cox proportional hazards model adjusting for demographics (age, sex), underlying comorbidities, clinical variable (body mass index), and laboratory measurements (serum D-dimers, serum ferritin, cTnI, WBCs, and C-reactive protein). The outcome discharge category was used to show survival, while the death category represented a hazard event. The Cox proportional hazards model showed an increased risk of death with high cTnI levels at admission. Patients with hypertension and diabetes together, history of ischemic heart disease, raised CRP, and D-dimer levels increased the risk of dying. While age, BMI, gender, hemoglobin (HB), serum ferritin, and WBCs did not affect the outcome (Table 3). We included a Kaplan-Meier survival probability plot for adjusted cTnI and comorbidities. Each plot represented the survival probabilities over time (Figures 1, 2).

**Table 3:** Cox proportional hazards regression model for age, BMI, gender, comorbidities, WBCs, CRP, ferritin, cardiac troponin I (cTnI), and D-dimers P<0.05; DM-diabetes mellitus; HTN-hypertension; WBCs-white blood cells; CRP-C-reactive proteins; BMI-body mass index; CI-confidence interval; HR-hazard ratio; B-unstandardized beta; SE-standard error; z-ratio of regression coefficient to its standard error

Variable	B	SE	95% CI	z	p	HR
Age	0.01	0.01	[-0.01,0.02]	0.88	.379	1.01
BMI	0.02	0.02	[-0.02,0.05]	0.88	.377	1.02
Gender (Female)	-0.01	0.15	[-0.31,0.28]	-0.10	.923	0.99
Hypertension	0.01	0.22	[-0.42,0.44]	0.05	.964	1.01
Diabetes Mellitus and Hypertension	1.42	0.28	[0.88,1.96]	5.14	< .001	4.14
Ischemic heart disease	0.62	0.22	[0.18,1.05]	2.78	.005	1.85
No Comorbidities	-1.46	0.42	[-2.28, -0.64]	-3.47	< .001	0.23
WBC Mildly Raised (B/w 11000 and 15000)	0.22	0.20	[-0.18,0.62]	1.08	.279	1.25
WBCs moderately raised (between 15000 and 20000)	0.23	0.22	[-0.21,0.67]	1.02	.308	1.26
WBCs severely raised (more than 20,000)	0.57	0.21	[0.16,0.98]	2.73	.006	1.77
CRP	0.87	0.31	[0.27,1.48]	2.84	.005	2.39
Ferritin high (more than 200)	-0.12	0.32	[-0.75,0.51]	-0.36	.716	0.89
Ferritin very high (more than 1000)	-0.52	0.29	[-1.10,0.05]	-1.79	.074	0.59
High troponin I (cTnI)	1.28	0.30	[0.70,1.86]	4.33	< .001	3.61
D-dimer high (more than 400)	1.02	0.42	[0.19,1.85]	2.40	.016	2.76
D-dimer very high (more than 1000)	1.55	0.38	[0.81,2.29]	4.11	< .001	4.70

## Discussion

The present study established a significant association between cardiac injury (detected by measuring cTnI) and outcome in hospitalized COVID-19 patients. To the best of our knowledge, this is the first and most extensive study to date in Pakistan investigating the impact of cardiac biomarkers on outcome in hospitalized COVID-19 patients. Our findings emphasized the inclusion of cardiac troponin I levels into everyday practice to detect, predict the severity of, and manage COVID-19 virus-infected patients. It has been shown to contribute to the COVID-19-related cardiac injury [3-6]. Male and elderly patients formed the bulk of our study group. The mean age (55.01 ± 13.49 years) of our research population was less than the mean age of patients in previous researches [6-8]. This trend showed a propensity of COVID-19 virus disease in young patients in this part of the world. The mean BMI was (24.94 ± 4.81), which was lower than the BMI of patients in previous studies [7-9]. The lower BMI suggested that patients in Pakistan infected by the SARS-CoV-2 virus were leaner than those in other parts of the world. Many studies have shown the rising mortality with higher BMI values [7-11]. While on the contrary, our research’s Cox regression analysis revealed that BMI did not affect the outcome (HR 1.02 [confidence interval (CI) 95%, -0.02,0.05], p=.377).

In our study, mortality was higher in patients with high cTnI levels (HR 3.61 [CI 95%, 0.70,1.86], p<.001), patients suffering from both hypertension and diabetes together (HR 4.14 [CI 95%, 0.88,1.96], p<.001), and in patients who had a history of ischemic heart disease (IHD) (HR 1.85 [CI 95%, 0.18,1.05], p=.005). These findings are in line with the findings of Santos et al. [12].

Viral diseases, such as influenza, can often present as cardiac injury. Several studies have endorsed this statement. In the 2009 H1N1 pandemic, retrospective findings showed that 54% of patients had cardiac insult [13]. Cases of myocarditis were recorded in 2012 by MERS-CoV [13-15]. Likewise, coronavirus disease (COVID-19) is a lethal infectious disease that has infected over 58.8 million people worldwide as of November 2020. It surfaced in Wuhan, China and has spread, resulting in a growing pandemic [16-18]. Given the rising incidence of COVID-19 virus disease, research in the domain was much needed. The rapid surge in COVID-19 demanded urgent attention both in the diagnostic and therapeutic contexts. This urgency led us to conduct this research on admitted patients of COVID-19 virus disease.

Multiple body organs are infected by COVID-19 virus disease [19]. Among these, cardiac involvement is not rare. Cardiac involvement has also been reflected in our research: 168 (36.05 %) patients have raised levels of cTnI. More than half of the patients in our sample died from a myocardial injury, consistent with the results of Yange et al., Pzizzini et al., and Gao et al. [17,19,20]. In contrast, 19% died with COVID-19 infection with raised cTnI level in a study by Shi et al. [21]. Our research included more patients with myocardial injury and elevated cTnI levels than previously published studies [16,22-26]. As evident in previous studies, the rate and quasi nature of abnormal cTnI levels in COVID-19-infected cases prompted clinicians to measure cTnI levels only when they suspected acute myocardial infarction. In comparison, we have tried to determine that all patients infected with COVID-19 should be screened for myocardial injury by carrying out cTnI levels. Our findings demonstrated that cTnI acted as a marker of severity in COVID-19 virus disease and was raised in around one-third of COVID-19 patients.

Multiple studies have examined the relationship between rising cardiac enzymes and poor prognosis in COVID-19 virus disease. These findings were in line with our research. The coefficient for unadjusted elevated cTnI levels was significant (HR 6.09 [CI 95%, 1.50,2.11], p<.001), showing that a rise in cTnI has a hazard that is 6.09 times as large as normal cTnI levels.

According to Huang et al., diabetes mellitus has increased mortality and disease severity in COVID-19 [27]. In our study, people with only diabetes as a sole risk factor did not have high mortality, while those with both diabetes and hypertension as their risk factors were at increase risk of dying (HR 4.14 [CI 95%, 0.88,1.96], p<.001).

In a study conducted by Nie et al., a total of 311 patients diagnosed with COVID-19 virus disease were enrolled. Among them, 111 patients died, and 200 survived. The cardiac injury was present in 103 (33.1%) patients. 12 of these patients were in the discharged category, and 91 were in the nonsurvivor category. Multivariable logistic regression was carried out, which declared cardiac troponin I as a predictor of mortality in COVID-19 patients [28]. In comparison, we conducted our study on 466 hospitalized patients, of which 200 died and 266 survived. cTnI was raised in 168 patients. 38 of them were in the discharge group, while 130 were in the nonsurvivor category. We conducted the Cox proportional hazards model, which showed an increased risk of dying in patients with high cTnI levels at admission, which is consistent with the findings of Nie et al. We did not go into details of the pathogenesis involved in each case. We only aimed to establish the fact that there is an association between cardiac markers, i.e., cTnI and disease severity in COVID-19 affected patients. 

Limitations

Our study had certain limitations; First, our study was a single-centred study. Second, we did not consider the underlying pathology of myocardial injury leading to a rise in cTnI levels. Third, we did not measure serial (cTnI) levels because of limited resources, which might have included or excluded patients. Our fourth limitation was that we did not consider all clinical and pathological confounders owing to the excessive patient burden, limited resources, and the risk of getting infected. We did not follow these patients after discharge from the hospital.

## Conclusions

The infection of COVID-19 is growing vigorously. With the rising number of new cases, awareness regarding its clinical presentations and diagnosis become more comprehensive. Cardiac biomarkers are typically elevated in patients with COVID-19 disease. The rise in cTnI can be explained by several mechanisms, from myocarditis to cytokine activity, inflicting myocardial injury. These mechanisms are controversial, and none has proved to be the main cause behind raised cTnI levels. Our study did not focus on the pathophysiology and concluded that hospitalized patients diagnosed with COVID-19 and raised cTnI are at increased risk of dying. In other words, high cTnI levels is an indicator of the severity and poorer outcome in patients with COVID-19 virus disease. Measuring cTnI levels in COVID-19 virus disease can direct physicians to triage patients and decrease mortality. Research on a larger scale is needed to drive a global reaction against COVID-19. Cardiac troponin elevations can be considered as a crude marker of severity. This has been illustrated in our study.
